# Investigation of High-Resolution Distributed Fiber Sensing System Embedded in Flexible Silicone Carpet for 2D Pressure Mapping

**DOI:** 10.3390/s22228800

**Published:** 2022-11-14

**Authors:** Zhanerke Katrenova, Shakhrizat Alisherov, Turar Abdol, Madina Yergibay, Zhanat Kappassov, Daniele Tosi, Carlo Molardi

**Affiliations:** 1 Department of Electrical and Computer Engineering, School of Engineering and Digital Sciences, Nazarbayev University, Astana 010000, Kazakhstan; 2 Department of Robotics Engineering, School of Engineering and Digital Sciences, Nazarbayev University, Astana 010000, Kazakhstan; 3 National Laboratory Astana, Laboratory of Biosensors and Bioinstruments, Kabanbay Batyr Ave, Astana 010000, Kazakhstan

**Keywords:** distributed fiber sensing, optical fibers, strain measurement, pressure sensing, 2D mapping

## Abstract

Fiber-optic sensors are a powerful tool to investigate physical properties like temperature, strain, and pressure. Such properties make these sensors interesting for many applications including biomedical applications. Fiber sensors are also a great platform for distributed sensing by using the principles of optical frequency domain reflectometry. Distributed sensing is becoming more and more used to achieve high-resolution measurements and to map physical properties of biomaterials at small scale, thus obtaining 2D and 3D mapping of a particular area of interest. This work aims at building and investigating a 2D sensing carpet based on a distributed fiber sensing technique, to map local pressure applied to the carpet. The two-dimensional mapping is obtained by embedding a single-mode optical fiber inside a soft silicone carpet. The fiber has been bent and arranged in a specific configuration characterized by several parallel lines. Different fiber fixation methods have been investigated by means of a comparative analysis to perform better characterization and to achieve a more precise response of the carpet. The best pressure sensitivity coefficient (0.373 pm/kPa or considering our setup 1.165 nm/kg) was detected when the fiber was fully embedded inside the silicone carpet. This paper demonstrates the possibility of mapping a 2D distributed pressure over a surface with a resolution of 2 mm by 2 mm. The surface of investigation is 2 cm by 6 cm, containing 310 sensing points. The sensing carpet has been validated selecting several preferential positions, by testing the consistency of the results over different portions of the carpet.

## 1. Introduction

In several technological applications, including biomedical applications, the role of fiber-optic sensors (FOS) is crucial. The growing interest in optical fiber sensors is given by their specific advantages with respect to other sensing technologies. In fact, FOS, specifically the ones exploiting glass optical fibers, present compact size, immunity to electromagnetic interference, biocompatibility, and the capability to be sterilized with standard medical sterilization approaches. These characteristics make FOS compatible for use in the biomedical field [1]. Furthermore, FOS are sensing devices that can provide measurements on several physical parameters, mainly temperature and strain, but other parameters such as pressure, force, and humidity can be also measured by exploiting the change in the properties of light propagating inside the optical fiber [2].

Various FOS technologies can be used to detect strain and temperature. Krohn et al. [3] divide FOS systems into three sensing families: point sensor, quasi-distributed sensors, and distributed sensors. The measurement of a particular parameter at a specific location is usually obtained with a point sensor. The main example of point sensor in optical fiber is the fiber Bragg grating (FBG). The FBG is a periodic modulation of the refractive index of the fiber core; it works like a notch filter for an optical signal, so that a broad band input light is transmitted through the FBG except for one specific wavelength, usually called Bragg wavelength, that is reflected. Temperature and strain variation can shift the Bragg wavelength, and that shift can be detected by a spectrometer. While FBG sensors represent a mature and reliable technology, they can be applied only locally, and the measurement refers to the location of the FBG [4,5]. The FBG technologies can be extended by multiplexing the FBGs in a single fiber, by inscribing an array of FBGs, each one of them with different Bragg wavelengths. In this case, it is possible to obtain a multi-point sensor, sometimes referred to quasi-distributed sensors. It is a great improvement with respect to the single FBG, since the sensor density is increased. On the other hand, there are limitations in terms of the numbers of FBGs in the array and in the physical distance between them. This caps the number of sensing points to a maximum of 40 per fiber and limits the spatial resolution of the sensors to roughly one centimeter [6,7]. A long distance between FBGs might reduce sensor accuracy and precision, which is critical in medical applications [1].

In order to achieve a larger number of sensing points, as well as a much narrow spatial resolution, it is possible to shift to distributed sensing technology. Optical frequency domain reflectometry (OFDR) is the key technique to achieve distributed sensing in optical fibers by enabling the interrogation of the weak backscattering signals that occurs during light-wave transmission [8,9]. The physical nature of optical fibers contains a backscattered signal of significantly lower intensity in comparison to the propagating light. The backscattering, namely Rayleigh scattering, presents a spectrum with chaotic fluctuations. On the other hand, the scattering is deterministic in nature, like a sort of fiber signature, and can be exploited for distributed sensing [8]. This spectral signature is sensitive to changes of temperature and strain, so that by correlating the backscattering signal to a reference it is possible to transform an entire single-mode optical fiber (SMF) in a distributed sensor, with great advantages in terms of spatial resolution that can be sub-millimeter [1,10]. The commercial implementation of OFDR principles is represented by the optical backscattering reflectometer (OBR) [11,12]. Compared with point sensors and quasi-distributed sensors, a distributed optical fiber sensor has peculiar characteristics that make it the most cost-effective tracking approach for various applications, including temperature mapping and shape sensing [13,14,15,16,17,18].

By obtaining multiple values at once, distributed sensing achieves highly resolved physical parameters such as temperature and strain. Tosi et al. [1] investigated the resolution of physical parameters at the millimeter-scale and showed the importance of distributed sensing techniques in biomedical applications. For instance, during minimally invasive thermal therapies, it is essential to obtain precise values in the temperature mapping of the ablated region [13]. The importance of getting narrow resolute values of physical parameters is also crucial for high-resolution diagnostics, needle and catheter tracking [19], and other additional new application fields such as microsurgery and ophthalmic procedures [20,21]. This approach can be extended to construct maps of strain, force, and pressure.

Previously, Roriz et al. [22] presented the advantages of FOS over conventional sensors in measuring strain and force in the biomechanical and biomedical fields. Constructing a map of applied pressure based on FOS will be a very attractive application for various biomedical areas, such as managing skin health during treatments in prosthetics and rehabilitation [23], and tactile sensing on detecting contact location and intensity in the human–robot interface [24]. To check human skin behavior, Kanellos et al. developed a flexible 2D pressure sensing surface based on FBG technology, measuring 12 MPa with a spatial resolution of 1 cm^2^ [25]. All of these applications rely on the use of multiple points sensors, such as arrays of FBGs, which permit simple demodulation but a resolution that is not always adequate. Some applications, like the measuring and mapping biting force, may require a substantially better resolution at millimeter scale.

In order to overcome the limitations, in this work, we report on developing a 2D sensing carpet based on a distributed sensing technique on a SMF. The two-dimensional approach is achieved by bending the optical fiber along the surface, creating parallel fiber lines inside the silicone carpet. By using OBR, a comparative analysis for various methods of fiber fixation inside the silicone material has been performed in order to evaluate the pressure sensitivity and to investigate the accuracy of the carpet. The sensing carpets under investigation present a large number of sensing points, for a maximum of 310, and can reconstruct the pressure map with a resolution of 2 mm by 2 mm (4 mm^2^).

## 2. Experimental Setup

### 2.1. Materials

In order to analyze the possibility of sensors based on distributed techniques and to determine the correlation between the distributed pressure and the local wavelength shift, the experiments were performed. To conduct these experiments and achieve point pressure sensing, a setup consisting of a rack structure and the pressing tool was designed in Solidworks. The rack structure and pressing tool were 3D printed with PLA (polylactic acid thermoplastic) on the Ultimaker S5 printer. The rack structure has two parts: a 200 mm by 140 mm table stand and a holder. The holder is cylindrical in shape so that the pressing tool could move vertically. The height of the holder is 21 mm, so that the pressing tool would not deviate while force measurements are taken.

**Figure 1:**
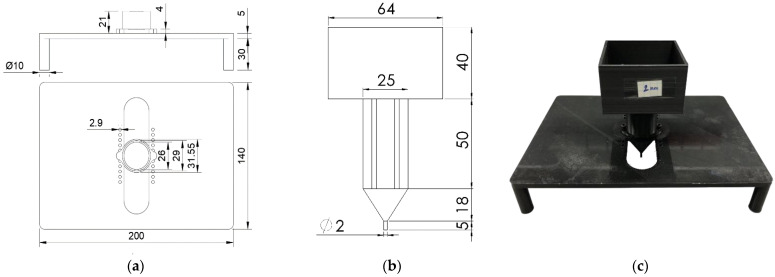
(**a**) Rack structure sketch: front and top view; (**b**) pressing tool sketch; (**c**) photograph of the rack structure.

The box dimensions of the pressing tool were designed to fit the calibration weights, which are 64 mm × 64 mm × 40 mm. The rounded cross-shaped extension of the box has a height of 50 mm, and it is then prolonged with an 18 mm cone that ends with a tip that has a height of 5 mm, as seen in Figure 1. For the experiment, the tips with diameters of 2 mm, 3 mm, and 4 mm were printed; however, only the 2 mm diameter tip was considered in this paper.

**Figure 2:**
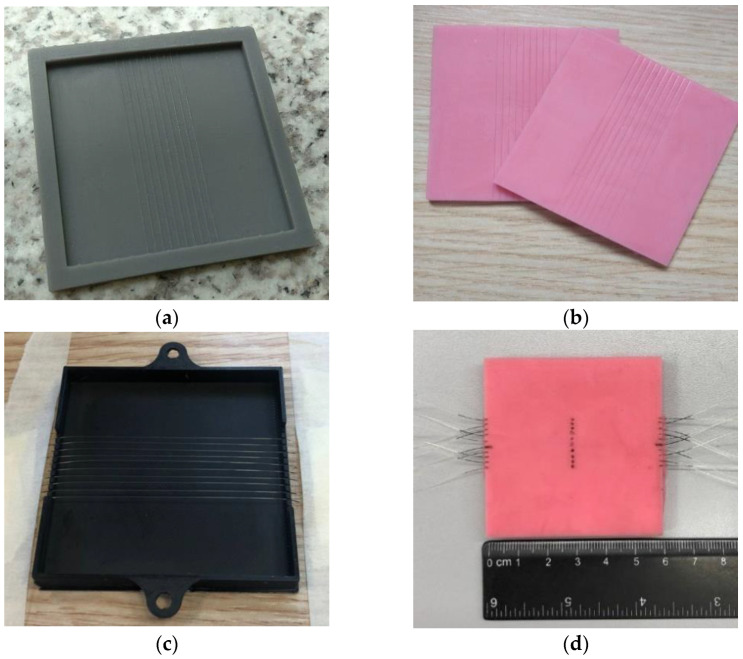
Preparation of the silicone with parallel grooves: (**a**) Grey V4 form for mold; (**b**) two prepared silicones; preparation of the embedded SMF in silicone: (**c**) the mold with fixed fiber lines on the working table; (**d**) embedded silicone prototype.

In order to conduct the experiments, two prototypes of 60 mm × 60 mm silicone models were prepared. The first prototype is the parallel fiber lines sandwiched between the silicone layers. Meanwhile, to achieve parallel configurations of fiber lines, the two silicone layers with parallel grooves were set up. Two 3 mm silicone sheets of this type were needed to create a fiber-embedded silicone for the experiment. The mold for this type was designed and 3D-printed on the Form 2 (Formlabs) printer with the Grey V4 material, as shown in Figure 2a. The steps for preparing the silicone with parallel grooves are as follows: two-component silicone compound Silcotin 20 with liquid-silicone A part and curing tin-catalyst B; part of the molding material is mixed in the ratio 50A:1B. As a result, a mixture with higher viscosity is created. If the ratio is incorrect, the material will have inconsistent density and Young’s modulus. Then, the bubbles from the mixture are removed with a vacuum pump. The mixture is poured into the Grey V4 mold with convex grooves, and, to accelerate the drying process, the silicone is placed into a UV oven for 120 min at 36 °C [26]. The mass of each silicone layer is 20 g, and the prepared layers are depicted in Figure 2b.

The second type of the silicone model is based on the fiber embedding in the silicone. The fiber-embedded silicone prototype was prepared by fixing fiber lines and by pouring the silicone mixture into the mold, as shown in Figure 2c. The mold is printed on the Ultimaker S5 printer with a PLA material.

The following steps were made to mold the silicones with embedded SMF. Firstly, the bottom part of the mold was fixed to the working table. The fiber was bent to the ten lines by fitting each line to holes on the sides of the mold. Secondly, the A and B parts of Silcotin 20 were mixed in the ratio 50A:1B, and bubbles were removed with the vacuum pump. Then, the mixture was poured into the mold with the fixed fibers and left on the table to cure for 24 h. The mass of the prepared silicone was 40 g, and the fiber embedded silicone is in Figure 2d. The Young modulus (E) of the Silcotin 20 material is defined by its nominal Shore toughness of 20, meaning E=0.73 MPa, according to Gent [27].

### 2.2. Methodology

A distributed sensing system can detect the spatial pattern of parameters such as strain and temperature along the optical fiber. Distributed sensing can be obtained by using optical frequency domain reflectometry technologies. The OFDR technique is a sophisticated and complex method to transform a standard and inexpensive single-mode fiber, like SMF-28 for telecommunication, into a flexible and powerful distributed sensor. In this context, Luna OBR is a practical implementation of OFDR. The OBR is capable of precisely detecting the backscattering spectrum, in a range of wavelengths from 1525 nm to 1610 nm, point by point, with the possibility of selecting the sensing point distance with a resolution that can be less than a millimeter. The backscattering properties of an optical fiber present a spectrum with chaotic characteristic. Even if the spectrum appears random, it is, instead, deterministic. For this reason, the detection of the backscattering, point by point, represents a sort of signature of the fiber. This spectral signature, like the Bragg wavelength of an FBG, shifts when a change of temperature or strain is applied. By saving the signature of the unperturbed fiber device, it is possible to cross-correlate this signature with the signature of the perturbed fiber. The obtained spectral shift is directly related to the local variation of temperature or strain [9].

In this work, the distributed strain detection is exploited. By applying different pressures in different positions over an elastic material that embeds an optical fiber, it is possible to detect a wavelength shift associated with a variation of strain along the fiber. The strain variation is given by the deformation of the elastic material under the pressure application. Since the position of the fiber inside the elastic material is known, the idea is to reconstruct a 2D pressure map, connected to the wavelength shift given by the local strain variation. With this methodology, a standard and inexpensive single-mode optical fiber can be exploited for a more complex and refined pressure detection.

**Figure 3:**
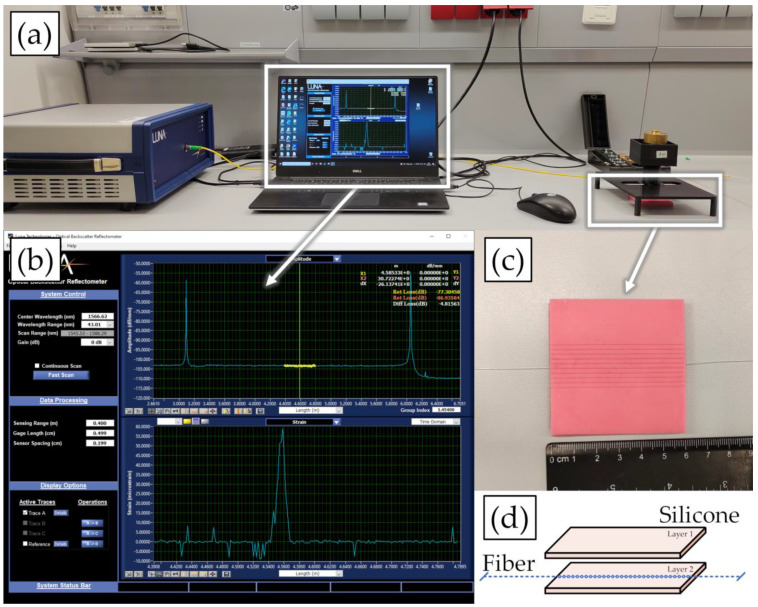
(**a**) Experimental setup. (**b**) The response on applied force. (**c**) One line fiber sandwiched between silicone layers. (**d**) Graphical representation of sandwiched fiber.

The working principle and experimental setup of the work are sketched in Figure 3. It includes Luna OBR4600, SMF, and its pigtail, calibration weights, a 3D-printed rack structure with a pressing tool, and silicone layers.

Table 1 represents the OBR parameters that were set up for the experiments, such as sensing range, gauge length, and sensor spacing. The sensing range of the wavelength-shift measurement was detected with respect to the length inside the silicone material, including the bending. The gauge length, which means the size of the sliding window, where the cross-correlation performance was obtained, was set to 0.5 cm; the sensor spacing shows the instrument’s capacity to resolve two nearby sensing locations. It was set to 0.2 cm. The OBR defines 310 strain sensing points based on the optical frequency-domain reflectometry concept and the previously set parameters.

As OBR measures the shift in the spectrum of the optical fiber, the reference of the untouched state was recorded for each measurement point.

Before beginning the measurements, the center of the rack structure was adjusted over the silicone with SMF inside it. After that, the sensing part was inserted into the round space with its tip accurately pressing on the measurement points at the chosen location on the silicone. The experiments were based on taking measurements by applying the different values of calibration weights to the selected points on the silicone material.

**Figure 4:**
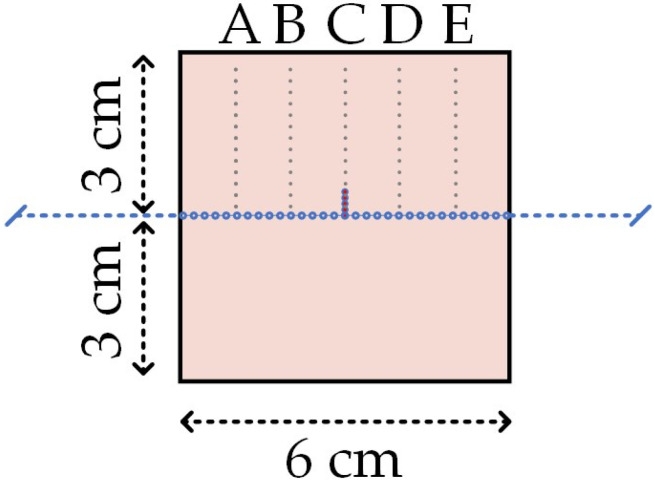
The schematic representation of the fiber on the silicone layer. The fiber line is divided in 5 points of investigation, spaced 1 cm between each other, tagged as A, B, C, D, E.

The calibration weights in the range of 50 to 700 g were manually put on top of the pressing tool. Consequently, the resultant difference in the spectrum, which is the principle of the distributed sensing approach, was saved and imported to MATLAB software for improved visualization. In order to obtain the optimal distance between fiber lines for parallel configurations, 500 g of weight was applied. The pressing tool with tip diameters of 2 mm, 3 mm, and 4 mm was adjusted at the upper points of the central position of the fiber line. The measured points are illustrated in Figure 4.

The preliminary results helped to determine and establish the distance between the fiber lines for achieving the specific configuration of fiber and getting the two-dimensional map of the applied pressure. The tip diameter was chosen at 2 mm to obtain precise results between parallel fiber lines at a distance of 2 mm. The calibration weights in the range of 50700 g was manually put on top of the pressing tool. Consequently, the resultant difference in the spectrum, which is the principle of the distributed sensing approach, was saved and imported to MATLAB software for improved visualization. In order to obtain the optimal distance between fiber lines for parallel configurations, 500 g of weight was applied. The pressing tool with tip diameters of 2 mm, 3 mm, and 4 mm was adjusted at the upper points of the central position of the fiber line. The measured points are illustrated in Figure 4. The preliminary results helped to determine and establish the distance between the fiber lines for achieving the specific configuration of fiber and getting the two-dimensional map of the applied pressure. The tip diameter was chosen at 2 mm to obtain precise results between parallel fiber lines at a distance of 2 mm.

In the next stage, after determining the optimal tip diameter, the wavelength-shift calibration against applied loads was performed for three different fiber fixation methods to analyze the pressure sensitivity [22]:

- Fiber sandwiched between silicone layers with no cut groove;
- Fiber sandwiched between silicone layers with a cut groove;
- Fiber embedded in the silicone.

In the first method, the fiber was sandwiched between two layers of silicone material with a thickness of 3 mm each. The second method was to put fiber into the groove that was cut manually. The printed groove was too wide for SMF. The fiber was fixed loosely, so the grooves were modified by cutting the lines with the stationary knife. The third method of fiber fixation is embedding the fiber in the silicone. The procedure for manufacturing the embedded technique was described in the previous section. The measurements were taken at the central point C above the fiber. It was chosen for follow-up observations since the results on the edge of the silicone layer were not consistent. As mentioned above, weights from 50 g to 700 g with a step of 50 g were attached to the tip to apply pressure to a given point in order to obtain pressure sensitivity for each fiber fixation method.

**Figure 5:**
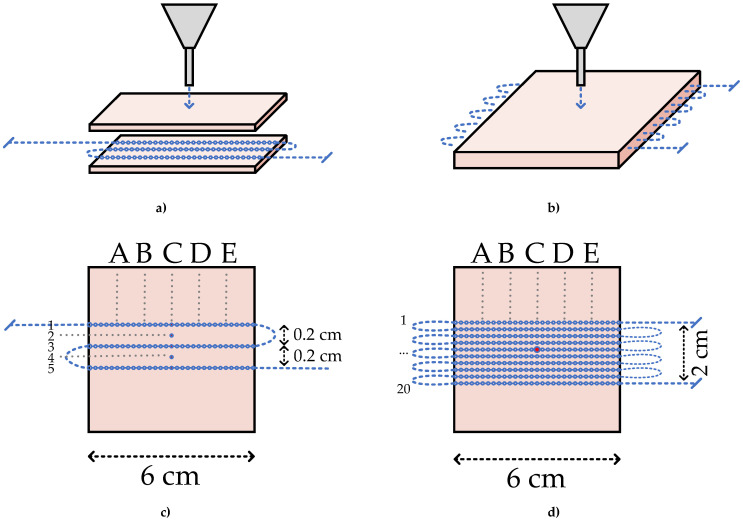
The fiber configurations for fiber sandwiched with cut silicone (**a**,**c**) and fiber embedded in the silicone fiber fixation method (**b**,**d**). Vertically, the fiber lines are divided in 5 points of investi-gation, spaced 1 cm between each other, tagged as A, B, C, D, E. Horizontally, the investigation lines are spaced of 1 mm, some of them positioned over fiber and some of them positioned in the middle. The investigation lines are tagged from 1 to 5 (**c**), and 1 to 20 (**d**).

In order to construct the two-dimensional approach, prototypes with the configuration of parallel fiber lines based on the fiber sandwiched with cut grooves and the fiber embedded in the silicone methods were prepared. Figure 5 illustrates the schematic representation of the prototypes. For the constructing matrix with the measured data, the columns correspond to the length of the silicone layer, with the sensing point at a distance of 2 mm, and the rows indicate bent parallel fiber lines at a distance of 2 mm. Thus, the final matrix comprised 31 points for every three rows along the column.

The first prototype with cut grooves had only three parallel fiber lines due to the challenge of bending the fiber. Due to an insufficient number of lines, the matrix was cropped. In order to build well and fully depict a 2D map of the final result, two additional zero lines were installed programmatically on both sides of the sensitive matrix of the given fiber for the fixation method. It should be noted that, due to manual cutting, the depth among fiber lines was inconsistent. The results of this method were demonstrated as a 0.8 × 6 cm^2^ 2D map. In the matrix with these above dimensions, the values of the wavelength shift were ignored in the bending region detected along the fiber lines. Since the fiber lines were not stable in the previous experiment, the manually cut grooves were not consistent and the number of lines was not enough for constructing a 2D map, it was decided to prepare the embedded prototype. In the given configuration, the fiber was bent into ten parallel fiber lines, thus obtaining a larger sensing surface with dimensions of 2 × 6 cm^2^ compared to the sandwiched fiber lines with the cut. Figure 5b,d depicts the embedded prototype with the coordinates on the sensing surface. The preparation of embedded fiber in silicone was explained previously in Section 2.1.

**Figure 6:**
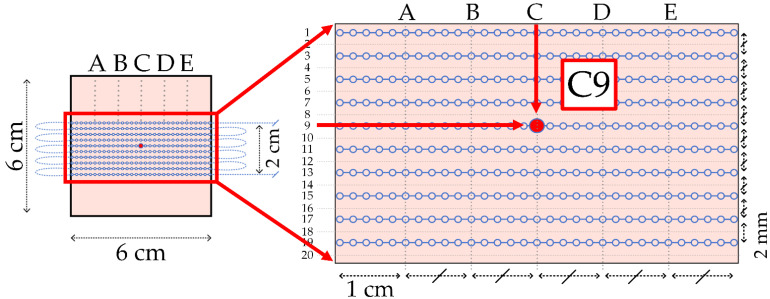
Graphical illustration of ten fiber lines embedded in silicone material. Vertically, the fiber lines are divided in 5 points of investigation, spaced 1 cm between each other, tagged as A, B, C, D, E. Horizontally, the investigation lines are spaced of 1 mm tagged from 1 to 20.

In contrast, the previous method had the fiber being removable from the model to efficiently test the same model instead of creating new ones for each experiment. Figure 6 illustrates the coordinates of the sensing points along the horizontal lines labeled as A, B, C, and D and the vertical lines indicated as digits from 1 to 20, the odd numbers for no fiber lines, and even numbers for ten parallel fiber lines in the silicone carpet.

## 3. Results and Discussion

### 3.1. Calibration

**Figure 7:**
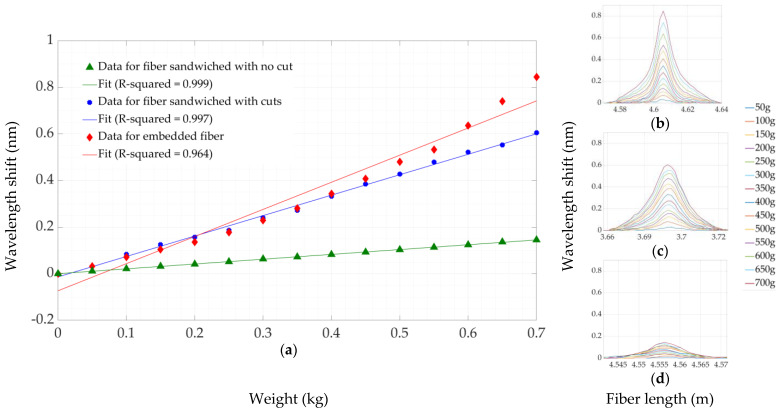
Peak wavelengths shift over applied weight (pressure sensitivity) of the three described fiber fixation methods (**a**). Wavelength shift distributed over the fiber in case of “embedded fiber” (**b**), “sandwiched fiber with cuts” (**c**), “sandwiched fiber without cuts” (**d**).

The calibration experiment was performed to ensure that the wavelength shift on the fiber was proportional to the applied loads. With the help of an experimental setup, including LUNA OBR, the measurements were taken with three different fiber fixation methods described above. Figure 7 shows a wavelength-shift curve, whose peaks are directly proportional to the values of weight, from 50 g to 700 g, with a step of 50 g. The graphs show that the highest response of wavelength shift was on embedded fiber in silicone material, thanks to the stringer fixation of fiber lines. Figure 7b–d illustrate the wavelength-shift dependence on the fiber fixation methods, such as the fiber embedded in silicone, the fiber sandwiched with cut, and the fiber sandwiched with no cut, respectively. The sensitivity curve was obtained by applying calibration weights from 50 g to 700 g to the three fiber fixation methods: green line for sandwiched fiber with no cut silicone, blue for the fiber sandwiched between silicone layers with cut, and red for the embedded fiber in the silicone material.

The sensitivities (wavelength shift over applied weight) are shown in Figure 7a. The sensitivity for the sandwiched case with no cut is 0.2068 nm/kg, equivalent to 0.0662 pm/kPa, since a tip diameter of 2 mm is not varied. The sensitivity for a sandwiched case with a cut in the silicone is 0.877 nm/kg, equivalent to 0.28 pm/kPa. Both these coefficients of sensitivity present a quite linear behavior with the r-squared values of 0.9998 and 0.9971. The case of fiber embedded in the silicone sheet, instead, presents a behavior that deviates from the linearity. Before an applied weight of 0.4 kg, the sensitivity is similar to the sandwiched case with cuts. After this value, the sensitivity increases with a nonlinear behavior. This nonlinearity is reflected in the sensitivity approximation; in fact, the linear approximation of the sensitivity is 1.165 nm/kg, equivalent to 0.373 pm/kPa, with r-squared values of 0.9637. This nonlinearly is possibly caused by the method of embedding. In the sandwiched case and in the sandwiched case with cuts, the fiber is not strictly bonded to the material; therefore, by applying the pressure over the silicone, the fiber receives a strain that is proportional to weight, and the response of the fiber depends on the rigidity of its material (glass). In the embedded case, the fiber is strictly bonded to the soft silicone substrate which is a material presenting nonlinearities in its elastic behaviors. Moreover, the embedded fiber is subjected to the internal tensions created by the solidification of the silicone during the molding of the sample. All of these effects translate into nonlinear strain sensitivity. In sandwiched fiber, despite having the best index of R-squared, 0.9998, the change in the strain value was not significant compared with embedded construction and the cut silicone method. The fiber embedding method has shown the best response to the applied loads in terms of reliable construction and has higher performance possibilities. The embedding of SMF straight into the model improves the accuracy of the performance of the experiments, permitting a reduction in the detrimental spread of the strain along the length of the fiber.

### 3.2. The Sandwiched Fiber with Cut Silicone Prototype

The wavelength-shift distribution among three bent fiber lines was inserted into the cuts. The results of the three central measurement points in two formats of different visualization using a calibration weight of 550 g were obtained. Referring to Figure 5c, “C1” is the sensing point on the upper fiber line, “C2” is the sensing point between upper and middle fiber lines, and “C3” is the sensing point on the middle fiber line.

**Figure 8:**
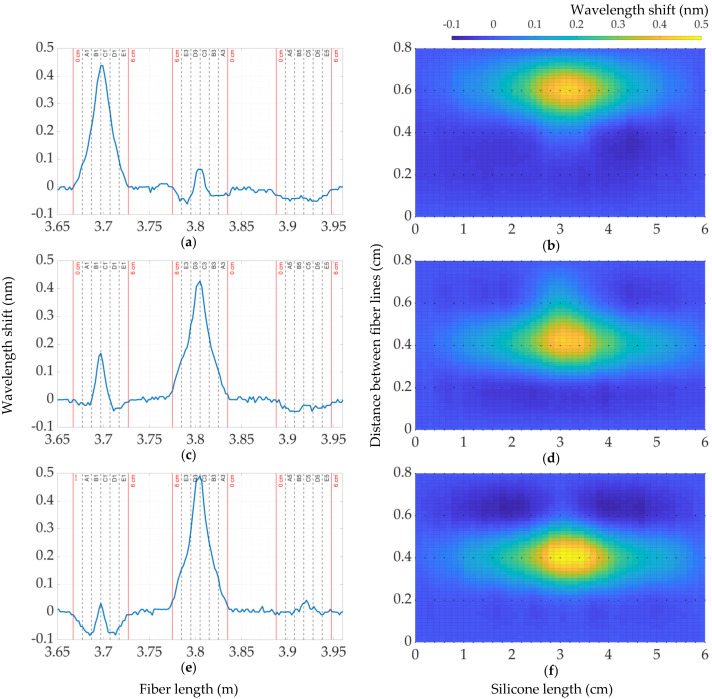
Plots and 2D maps of a wavelength shift to 550 g load: (**a**,**b**) position “C1”; **(c**,**d**) position “C2”; (**e**,**f**) position “C3”.

To begin with, Figure 8a was constructed using a fiber length of “C1” with respect to its wavelength shift. However, it is limited to the visualization of the whole fiber length, including the unnecessary intervals of bending. As is seen, the highlights are the intervals of each fiber line whose relationship between each other needs to be read. Instead, the length of each fiber line was set with respect to silicone in 2D maps, such as Figure 8b for “C1”. This reconstruction was done with the help of cubic spline interpolation that obtained the intermediate peaks between each fiber line. There were additional imaginary zero-filled vector lines on each side that improved the general map of interpolation.

On “C1”, with the applied load on this measurement point, the main fiber line was observed to have 0.4381 nm, while the closest neighboring line showed a significantly lower response, which was 0.06372 nm. The third fiber line, presenting a negative shift, shows a negative strain, which can be the result of a compression occurring for the bad placement of the fiber in the cut. Another observation was the elongation of strain along the whole fiber lines inside the silicone model. This distortion of stretching happened due to the fiber moving freely under the applied load.

Similarly, the alternative visualization of Figure 8c for “C2” is on the corresponding Figure 8d, where wavelength-shift distribution between first and second fiber lines are 0.1671 nm and 0.4268 nm, respectively. There is more strain in one line than the other due to the uneven manual cut having an effect on strain loss. Meanwhile, the remaining plot and 2D map for point “C3” display results from when the pressing tool was applied on the second fiber line that peaked at 0.4906 nm.

### 3.3. The Prortype of Fiber Embedding in the Silicone Material

**Figure 9:**
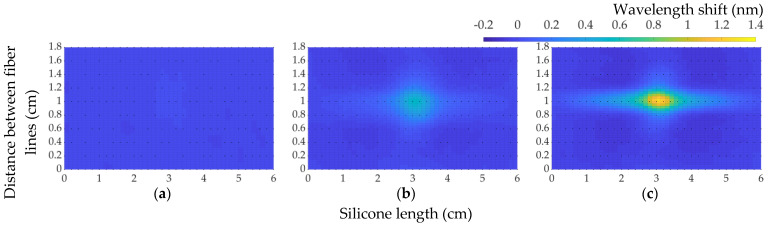
2D map of wavelength shift distribution on 50 g (**a**), 550 g (**b**), and 900 g (**c**).

The second configuration of the experimental prototype was performed on embedded fiber in the silicone material. In order to improve the limitations of the first prototype of cut silicone and increase the sensing area, described in previous sections, we 3D-printed a new mold for preparing a silicone sensing carpet with ten embedded fiber lines inside. The size of the first prototype was used as a base, and the fiber lines were fixed on the mold and filled with silicone, with a thickness of 3 mm on both sides. Thus, the sensing area was increased by 2 by 6 cm^2^. This part of the work is more explained in the Materials and Methodology sections. The sensing range on LUNA OBR was set as 1.6 m for obtaining whole fiber lines, including bending regions. Nevertheless, the regions where the fiber has been bent to configure the embedded ten sensing lines will be neglected during data processing. To demonstrate the weight sensitivity of the fiber-embedded silicone carpet was chosen the central sensing point “C9”, which corresponds to the fiber line located at a distance of 1 cm on the Y- axis respect to Figure 6. In Figure 9a–c are shown 2D maps of the distribution of wavelength shifts applying 50 g, 550 g, and 900 g, respectively.

**Figure 10:**
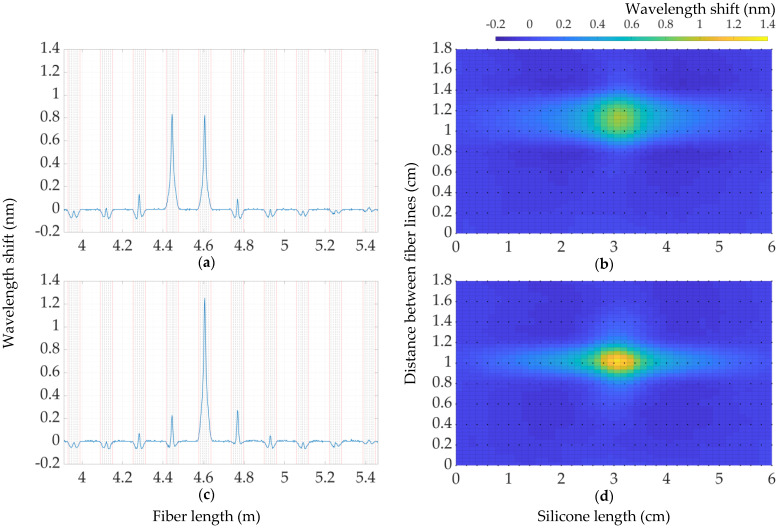
The distribution of a wavelength shift along the fiber on the points “C8” (**a,b**) and “C9” (**c,d**) for 900 g.

The next step was to take measurements on the central points of the silicone carpet to observe the distribution of the wavelength shift along the fiber. In Figure 10 are shown the distribution of the wavelength shift of an applied 900 g weight on the coordinates “C8” (Figure 10a,b) and “C9” (Figure 10c,d).

The left graphs represent the wavelength shift on the fiber length, and the red vertical lines show the sections of the embedded fibers, while the intervals between these red lines are bending regions, which are ignored for building the 2D map. As a result, in the right part of Figure 10 is demonstrated the 2D map corresponding to responses on sensing points “C8” and “C9”, respectively. In accordance with Figure 10a,b, it can be noticed that the response of a wavelength shift under the same weight on the coordinate “C8”, which is located between the two fiber lines (distance between “1” and “1.2” cm on the Y-axis of Figure 10b) is almost half of the response applied on the coordinate “C9” exactly along the fiber line, which are 0.83 nm and 1.25 nm, respectively. In view of the fact that the wavelength-shift response has a uniform distribution between the two fiber lines, the 2D map on the coordinate “C8” has the shape of a circle, while the distribution exactly on the fiber line has an ellipsoidal shape. However, since the applied weight was the same in both cases, the response on the point between fiber lines should also be similar to the response along the fiber, and this should be applicable to other points no matter the coordinates of applying weights. In this regard, it is necessary to perform the post-processing of data or to improve the performance of the prototype, but this part is not covered in this paper and may be improved in future work. Eventually, it is worth noting that every embedded fiber line presents a sort of ripple when not directly involved in the pressure detection. This ripple appeared to be the consequences of the internal tension induced during the process of molding the silicone around the embedded fiber.

**Figure 11:**
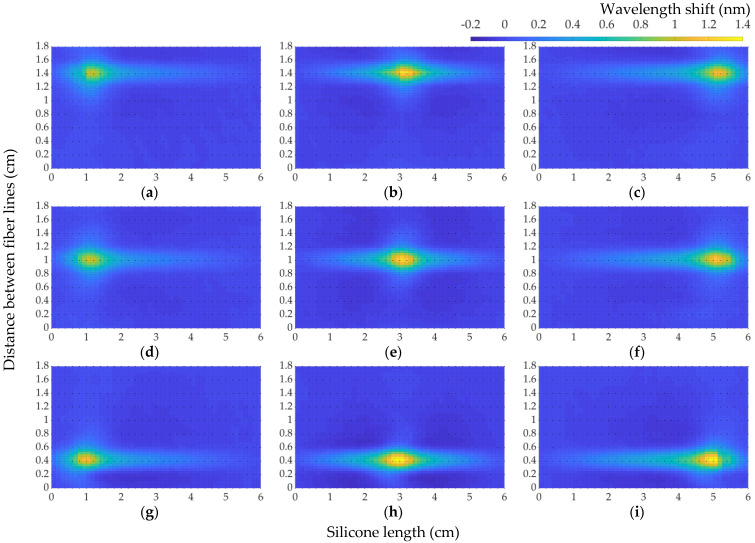
The measurements on wavelength shift over the embedded fiber in silicone carpet: (**a**) “A5”, (**b**) “C5”, (**c**) “E5”, (**d**) “A9”, (**e**) “C9”, (**f**) “E9”, (**g**) “A15”, (**h**) “C15”, and (**i**) “E15”.

In order to check the fabrication quality of the sensing carpet based on embedding method nine, spatially scattered locations were chosen. Figure 11 represents the wavelength-shift variation of the chosen coordinates along the sensing map. The coordinates “A5”, “C5”, and “E5” were chosen for the measuring of the upper side of the sensing map, and “A9”, “C9”, and “E9” were chosen for the middle side of the carpet, and, for the lower part of the sensing surface, “A15”, “C15”, and “E15” were chosen. The names of coordinates were depicted previously in Figure 6. The measurements were taken on a weight of 900 g. The measurement points were chosen on the third, fifth, and ninth fiber lines to obtain an overall picture of the sensing carpet.

As can be seen in Figure 11, despite the fact that the same weight (900 g) was applied, the wavelength-shift value varied from point to point. The left part of the sensing carpet has been less sensitive to the applied weight. Such variable results along the different part of the surface were obtained due to the fact that, during the embedding of the fiber in the silicone material, each fiber line was fixed to the mold separately and manually. The depth of the fixed fiber lines or the position of the parallel fiber lines could be shifted a little bit during the embedding process.

Nevertheless, the variance in the wavelength-shift values of the chosen coordinates is low, 0.0168. Table 2 represents the statistical values of the wavelength shift for nine locations on the sensing carpet.

## 4. Conclusions

In conclusion, we reported the fabrication of a two-dimensional sensing carpet based on a distributed fiber sensing technique. The 2D map concept was reached by bending fiber in ten lines. Different methods were investigated, such as sandwiched fiber with no cut, sandwiched fiber with cut silicone, and the embedded fiber in the silicone. An analysis of the fiber fixation method has been reported. Calibration steps were done by applying weights from 50 g to 700 g. The sensitivity curve coefficients of fiber sandwiched with no cut is 0.2068 nm/kg, fiber sandwiched with cut silicone is 0.877 nm/kg, and fiber-embedded silicone is 1.165 nm/kg with r-squared values of 0.9998, 0.9971, and 0.9637, respectively. The final prototype—fiber-embedded silicone carpet—was validated on weights up to 1 kg. The 2D sensing map was prepared by constructing the matrix and using interpolation for the collected data on each fiber line. The detection of wavelength-shift values caused by the variation of backscattered power on actual fiber locations was constructed on a two-dimensional sensing carpet. The uniqueness of sensing surfaces based on their mechanical characteristics allows for greater response to curvature by embedding or attaching the sensor to irregular forms and geometries, providing better reaction to curvature.

The proposed sensing surface with 310 sensing points is easily extendable and can be used as a fundamental part for many other sensors, according to the field of their application. On the other hand, the nature of the distributed sensing system, the large amount and the density of the sensing points suggest a more suitable set of applications in the biomedical field. A distributed pressure-sensing carpet can be of interest in prosthetics and rehabilitation field for controlling skin health and preventing the onset of pressure ulcers in patients. Similarly, a small and surface-adaptable pressure sensor can be used in soft robotic applications for functional compensation or to develop a robot–machine interface for tactile sensing wherein the simultaneous detection of location and intensity of pressure is usually challenging. Another possible application is related to the orthodontics field in order to map the bite force. In this application, it is fundamental to increase the density of sensing points to achieve a precise pressure map, to detect the possible malocclusion of the jaw.

Further extension of this research will be improved for constructing a two-dimensional map of pressure concerning the shape of a bite for a mouthguard. It can also be improved using an alternative configuration using nanoparticles-doped scattering-level multiplexing to set up multiple fiber lines.

## Figures and Tables

**Table 1:** System parameters.

Center Wavelength (nm)	Wavelength Range (nm)	Sensing Range (m)	Gage Length (cm)	Sensor Spacing (cm)	Spatial Resolution (cm)
1566.62	43.01	0.5 ^1^	0.5	0.2	0.1

^1^ May depend on the measuring range of the fiber.

**Table 2:** The statistical values of the chosen points of the sensing carpet.

Coordinates/Wavelength Shift (nm)	“A”	“C”	“E”
“5”	0.98	1.33	1.1
“9”	1.1	1.2	1.2
“15”	1.11	1.38	1.36
Mean, μ		1.195	
Standard deviation, σ		0.1296	
Variance, $\sigma^{2}$		0.0168	
Min		0.98	
Max		1.38	

## Data Availability

Data presented in this work are not publicly available at this time. Raw data can be obtained upon reasonable request from the authors.

## References

[1] Tosi D., Schena E., Molardi C., Korganbayev S. (2018). Fiber optic sensors for sub-centimeter spatially resolved measurements: Review and biomedical applications. Opt. Fiber Technol..

[2] Udd E., Spillman W. (2011). Fiber Optic Sensors: An Introduction for Engineers and Scientists.

[3] Krohn D.A., MacDougall T., Mendez A. (2014). Fiber Optic Sensors: Fundamentals and Applications.

[4] Erdogan T. (1997). Fiber Grating Spectra. J. Light. Technol..

[5] Rao Y. (1999). Recent progress in applications of in-fibre Bragg grating sensors. Opt. Lasers Eng..

[6] Li C., Tang J., Cheng C., Cai L., Yang M. (2021). FBG Arrays for quasi-distributed sensing: A review. Photon. Sens..

[7] Lindner E., Hartung A., Hoh D., Chojetzki C., Schuster K., Bierlich J., Rothhardt M. (2014). Trends and future of fiber Bragg grating sensing technologies: Tailored draw tower gratings (DTGs). Optical Sensing and Detection III.

[8] Bao X., Chen L. (2012). Recent progress in distributed fiber optic sensors. Sensors.

[9] Froggatt M., Moore J. (1998). High-spatial-resolution distributed strain measurement in optical fiber with Rayleigh scatter. Appl. Opt..

[10] Kreger S.T., Rahim N.A.A., Garg N., Klute S.M., Metrey D.R., Beaty N., Jeans J.W., Gamber R. Optical frequency domain reflectometry: Principles and applications in fiber optic sensing. Proceedings of the SPIE Commercial + Scientific Sensing and Imaging.

[11] OBR 4600 Optical Backscatter Reflectometer TM. https://lunainc.com/product/obr-4600.

[12] Soller B.J., Gifford D.K., Wolfe M.S., Froggatt M.E. (2005). High resolution optical frequency domain reflectometry for characterization of components and assemblies. Opt. Express.

[13] Schena E., Tosi D., Saccomandi P., Lewis E., Kim T. (2016). Fiber optic sensors for temperature monitoring during thermal treatments: An overview. Sensors.

[14] Ashikbayeva Z., Aitkulov A., Jelbuldina M., Issatayeva A., Beisenova A., Molardi C., Saccomandi P., Blanc W., Inglezakis V., Tosi D. (2020). Distributed 2D temperature sensing during nanoparticles assisted laser ablation by means of high-scattering fiber sensors. Sci. Rep..

[15] Beisenova A., Issatayeva A., Sovetov S., Korganbayev S., Jelbuldina M., Ashikbayeva Z., Blanc W., Schena E., Sales S., Molardi C. (2019). Multi-fiber distributed thermal profiling of minimally invasive thermal ablation with scattering-level multiplexing in MgO-Doped fibers. Biomed. Opt. Express.

[16] Issatayeva A., Amantayeva A., Blanc W., Tosi D., Molardi C. (2021). Design and analysis of a fiber-optic sensing system for shape reconstruction of a minimally invasive surgical needle. Sci. Rep..

[17] Parent F., Loranger S., Mandal K.K., Iezzi V.L., Lapointe J., Boisvert J.-S., Baiad M.D., Kadoury S., Kashyap R. (2017). Enhancement of accuracy in shape sensing of surgical needles using optical frequency domain reflectometry in optical fibers. Biomed. Opt. Express.

[18] Miller G.A., Askins C.G., Friebele E.J. (2004). Shape Sensing Using Distributed Fiber Optic Strain Measurements. Proceedings of the Second European Workshop on Optical Fibre Sensors.

[19] Liu H., Farvardin A., Pedram S.A., Iordachita I., Taylor R.H., Armand M. (2015). Large Deflection Shape Sensing of a Continuum Manipulator for Minimally-Invasive Surgery. Proceedings of the 2015 IEEE International Conference on Robotics and Automation.

[20] Gonenc B., Chae J., Gehlbach P., Taylor R.H., Iordachita I. (2017). Towards robot-assisted retinal vein cannulation: A motorized force-sensing microneedle integrated with a handheld micromanipulator. Sensors.

[21] Mandal K., Parent F., Martel S., Kashyap R., Kadoury S. (2016). Vessel-based registration of an optical shape sensing catheter for MR navigation. Int. J. Comput. Assist. Radiol. Surg..

[22] Roriz P., Carvalho L., Frazão O., Santos J.L., Simões J.A. (2014). From conventional sensors to fibre optic sensors for strain and force measurements in biomechanics applications: A review. J. Biomech..

[23] Tsiokos D., Kanellos G.T., Papaioannou G., Pissadakis S., Hudak R., Penhaker M., Majernik J. (2012). Fiber Optic-Based Pressure Sensing Surface for Skin Health Management in Prosthetic and Rehabilitation Interventions. Biomedical Engineering.

[24] Massari L., Fransvea G., D’Abbraccio J., Filosa M., Terruso G., Aliperta A., D’Alesio G., Zaltieri M., Schena E., Palermo E. (2022). Functional mimicry of Ruffini receptors with fibre Bragg gratings and deep neural networks enables a bio-inspired large-area tactile-sensitive skin. Nat. Mach. Intell..

[25] Kanellos G.T., Papaioannou G., Tsiokos D., Mitrogiannis C., Nianios G., Pleros N. (2009). Two dimensional polymer-embedded quasi-distributed FBG pressure sensor for biomedical applications. Opt. Express.

[26] Luis E., Pan H.M., Sing S.L., Bastola A.K., Goh G.D., Goh G.L., Tan H.K.J., Bajpai R., Song J., Yeong W.Y. (2019). Silicone 3D printing: Process optimization, product biocompatibility, and reliability of silicone meniscus implants. 3D Print. Addit. Manuf..

[27] Gent A.N. (1958). On the relation between indentation hardness and Young’s modulus. Rubber Chem. Technol..

